# Absence of Bsep/Abcb11 attenuates MCD diet‐induced hepatic steatosis but aggravates inflammation in mice

**DOI:** 10.1111/liv.14423

**Published:** 2020-03-18

**Authors:** Claudia D. Fuchs, Sebastian Krivanec, Daniel Steinacher, Veronika Mlitz, Annika Wahlström, Marcus Stahlman, Thierry Claudel, Hubert Scharnagl, Tatjana Stojakovic, Hanns‐Ulrich Marschall, Michael Trauner

**Affiliations:** ^1^ Hans Popper Laboratory of Molecular Hepatology Division of Gastroenterology and Hepatology Department of Internal Medicine III Medical University of Vienna Vienna Austria; ^2^ Department of Molecular and Clinical Medicine Wallenberg Laboratory Institute of Medicine Sahlgrenska Academy University of Gothenburg Gothenburg Sweden; ^3^ Clinical Institute of Medical and Chemical Laboratory Diagnostics Medical University of Graz Graz Austria; ^4^ Clinical Institute of Medical and Chemical Laboratory Diagnostics University Hospital Graz Graz Austria

**Keywords:** bile acid metabolism, FXR signaling, PPARα signaling

## Abstract

**Background:**

Bile acids (BAs) regulate hepatic lipid metabolism and inflammation. Bile salt export pump (BSEP) KO mice are metabolically preconditioned with a hydrophilic BA composition protecting them from cholestasis. We hypothesize that changes in hepatic BA profile and subsequent changes in BA signalling may critically determine the susceptibility to steatohepatitis.

**Methods:**

Wild‐type (WT) and BSEP KO mice were challenged with methionine choline‐deficient (MCD) diet to induce steatohepatitis. Serum biochemistry, lipid profiling as well as intestinal lipid absorption were assessed. Markers of inflammation, fibrosis, lipid and BA metabolism were analysed. Hepatic and faecal BA profile as well as serum levels of the BA synthesis intermediate 7‐hydroxy‐4‐cholesten‐3‐one (C4) were also investigated.

**Results:**

Bile salt export pump KO MCD‐fed mice developed less steatosis but more inflammation than WT mice. Intestinal neutral lipid levels were reduced in BSEP KO mice at baseline and under MCD conditions. Faecal non‐esterified fatty acid concentrations at baseline and under MCD diet were markedly elevated in BSEP KO compared to WT mice. Serum liver enzymes and hepatic expression of inflammatory markers were increased in MCD‐fed BSEP KO animals. PPARα protein levels were reduced in BSEP KO mice. Accordingly, PPARα downstream targets Fabp1 and Fatp5 were repressed, while NFκB subunits were increased in MCD‐fed BSEP KO mice. Farnesoid X receptor (FXR) protein levels were reduced in MCD‐fed BSEP KO vs WT mice. Hepatic BA profile revealed elevated levels of TβMCA, exerting FXR antagonistic action, while concentrations of TCA (FXR agonistic function) were reduced.

**Conclusion:**

Presence of hydroxylated BAs result in increased faecal FA excretion and reduced hepatic lipid accumulation. This aggravates development of MCD diet‐induced hepatitis potentially by decreasing FXR and PPARα signalling.

**Abbreviations**
ALT: alanine aminotransferase
AP: alkaline Phosphatase
AST: aspartate aminotransferase
BA: bile acid
BSEP/ABCB11: bile salt export pump
C4: 7‐hydroxy‐4‐cholesten‐3‐one
CA: cholic acid
CDCA: chenodeoxycholic acid
COL1a1: collagen 1a1
COL1a2: collagen 1a2
CYP3a11: cytochrome P450, family 3, subfamily a, polypeptide 11
CYP7A1: cholesterol 7 alpha‐hydroxylase
DCA: deoxycholic acid
DGAT2: diglyceride acyltransferase
ErDj4: endoplasmic reticulum–localized DnaJ 4
Fabp1: fatty acid binding protein 1
Fatp5: fatty acid transport protein 5
FFA: free fatty acids
FXR: Farnesoid X receptor
GRP78: glucose regulated protein 78kDa
IHC: immunohistochemistry
LCA: lithocholic acid
MCA: muricholic acid
MCD: methionine choline‐deficient
MCP1: monocyte chemotactic protein 1
NAFLD: non‐alcoholic fatty liver disease
NASH: non‐steatohepatitis
PHBAs: polyhydroxylated bile acids
SCD1: stearoyl‐CoA desaturase
SHP: short heteromer partner
SREBP1c: sterol response element binding protein 1c
TG: triglyceride
TLCA: taurolithocholic acid
TNFα: tumour necrosis factor alpha
UDCA: ursodeoxycholic acid
WT: wild‐type
αSMA: alpha smooth muscle actin

**Key points**

Metabolic precondition with a hydrophilic BA pool results in the dissection of steatosis and inflammation development in a mouse model of steatohepatitis. While steatosis was attenuated inflammation was aggravated.

## 1 INTRODUCTION

Non‐alcoholic fatty liver disease (NAFLD) comprises a wide disease spectrum ranging from simple steatosis to steatohepatitis (NASH), fibrosis, cirrhosis and cancer.^1^, ^2^, ^3^ The mechanisms underlying the progression from benign steatosis to NASH and more advanced disease stages are still poorly understood. Free fatty acids (FAs), especially saturated fatty acids, were proposed to act as lipotoxic triggers,^4^ driving disease progression from steatosis to NASH. However, polyunsaturated FAs also serve as ligands for PPARα,^5^ which correlate negatively with the severity of NASH in humans.^6^ Accordingly, a beneficial role of PPARα and PPARδ agonists has been demonstrated in several (pre)clinical NAFLD/NASH studies.^7^, ^8^, ^9^, ^10^, ^11^ Moreover, bile acids (BAs), via signalling through their dedicated nuclear receptor farnesoid X receptor (FXR; NR1H4) as key regulator of glucose and lipid metabolism, as well as inflammation^12^, ^13^, ^14^, ^15^, ^16^, ^17^ may play an important role in the pathogenesis and treatment of NAFLD/NASH. FXR KO mice exert decreased insulin sensitivity and have a pro‐atherogenic lipoprotein profile with substantially elevated serum and hepatic cholesterol and TG levels.^17^ The severity of NAFLD/NASH in humans has also been linked to reduction of FXR signalling^18^, ^19^ and changes in BA levels and composition.^20^ Conversely, pharmacological activation of FXR is beneficial in patients with NAFLD and NASH.^21^

Bile acids are excreted from hepatocytes into bile by the bile salt export pump (BSEP, ABCB11).^22^, ^23^ Notably, associations between BSEP variants and increased serum triglycerides (TG)^24^ as well as cholesterol^24^, ^25^ and obesity^26^ have been reported in humans. In line, mice overexpressing BSEP display reduced hepatic steatosis when fed a lithogenic diet^27^ or a methionine choline‐deficient (MCD) diet.^28^ In the present study, we aimed to explore how increased BA hydroxylation and subsequent changes in BA signalling, only seen in BSEP KO mice conferring protection against cholestatic liver injury,^29^ may impact on development of fatty liver and progression to steatohepatitis.

Therefore, wild‐type (WT) and BSEP KO mice were subjected to MCD diet, as a model of hepatic steatosis associated with profound inflammation.

## 2 MATERIALS AND METHODS

### 2.1 Animal experiments

Male BSEP KO and WT FVB/N mice were kindly provided by the British Columbia Cancer Research Center.^30^ Age‐matched animals were fed a MCD diet (obtained from SAFE—Scientific Animal Food & Engineering) for 5 weeks ad libitum. This animal study was approved by the Animal Ethics Committee of the Medical University of Vienna and the Federal Ministry of Science, Research and Economy (BMWFW‐66.010/0089II/3b/2010) and was performed according to the Animal Research: Reporting of In Vivo Experiments (ARRIVE) guidelines.

### 2.2 Serum biochemistry

Blood was collected at harvesting and centrifuged for 20 minutes at 1500 *g*. Serum was stored at −80°C until analysis. Levels of transaminases (aspartate aminotransferase, AST; alanine aminotransferase, ALT), alkaline phosphatase (AP), total cholesterol, TG (Roche Diagnostics, Mannheim, Germany), FAs (Wako Chemicals GmbH, Neuss, Germany) and BAs (DiaSys Diagnostic Systems GmbH, Holzheim, Germany) were measured using enzymatic methods according to the manufacturer's instructions. High‐, low‐ and very low‐density lipoprotein (HDL, LDL and VLDL) cholesterol were assessed by quantitative agarose gel electrophoresis (Helena Biosciences, Gateshead, UK).

### 2.3 Liver histology

For conventional light microscopy, livers were fixed in 4% neutral‐buffered formaldehyde solution for 24 hours and embedded in paraffin. Sections were cut 4 µm thick and stained with haematoxylin and eosin and Sirius Red.

### 2.4 Immunohistochemistry for F4/80

To quantify and characterize the hepatic inflammatory cell infiltrate, immunohistochemistry for F4/80^+^ (F4/80 IHC) cells was performed on formaldehyde (4% neutral‐buffered)‐fixed, paraffin‐embedded liver sections using a monoclonal mouse antibody. Sections were deparaffinated, rehydrated and digested with 0.1% protease. Endogenous peroxidase was blocked with 1% H_2_O_2_ in methanol. Specific binding of the F4/80 antibody was detected using a biotinylated anti‐rat IgG and the ABC‐System with amino‐9‐ethyl‐carbazole as substrate.^31^ Antibody details: F4/80 Monoclonal Antibody (BM8), eBioscience™, catalog # 14‐4801‐82, Thermo Fisher Scientific.

### 2.5 Messenger RNA analysis and real‐time quantitative polymerase chain reaction

RNA isolation, complementary DNA synthesis and real‐time quantitative PCR were performed according to the manufacturer’s instructions. All data were normalized to 36b4 and shown as mean ± SD. All oligonucleotide sequences are listed in Table S1.

### 2.6 Western blotting

Protein isolation (nuclear extracts for FXR, PPARα and tubulin; total protein for αSMA and β‐actin) and western blotting were performed.^32^ Antibody details: FXR: NR1H4 antibody (EPR5721), catalog # ab126602, abcam; PPARα: PPARα antibody (H‐98), catalog # sc‐9000, Santa Cruz; Tubulin: Tubulin antibody clone B‐5‐1‐2, catalog # T5168, Sigma‐Aldrich; αSMA: α Smooth Muscle Actin antibody clone 1A4, catalog # A2547, Sigma‐Aldrich; βActin: β‐Actin (8H10D10) antibody, catalog # 3700, Cell Signaling.

### 2.7 Hepatic lipid content

Lipids were extracted by a methyl tert‐butyl ether (MTBE) protocol as previously described.^33^ Briefly, liver pieces were extracted in methanol/MTBE/water and the organic phase was dried, resuspended in 500 µL methanol/chloroform 1:1 and 12:0/13:0 PC, 17:0/20:4 PC, 14:1/17:0 PC, 21:0/22:6 PC, 17:1 LPC, TG‐Mix LM6000 (1.25 µmol/L each), 12:0/13:0 PS, 17:0/20:4 PS, 14:1/17:0 PS, 21:0/22:6 PS, DG‐Mix LM6001 (3 µmol/L each), 12:0/13:0 PE, 17:0/20:4 PE, 14:1/17:0 PE, 21:0/22:6 PE, 12:0/13:0 PI, 17:0/20:4 PI, 14:1/17:0 PI and 21:0/22:6 PI (2 µmol/L each) were added as internal standard. An 50 µL aliquot thereof was again dried and resuspended in 90 µL isopropanol:chloroform:methanol (90:5:5 v/v/v). Data acquisition was performed on an LTQ Orbitrap Velos Pro instrument (Thermo Scientific) coupled to a Dionex Ultimate 3000 UHPLC (Thermo Scientific) according to previously published protocols.^34^, ^35^ In a nutshell, chromatographic separation was performed on a Waters (Waters, Milford, MA, USA) BEH C8 column (100 × 1 mm, 1.7 μm), thermostatted to 50°C. Mobile phase A was deionized water containing 1 vol% of 1 mol/L aqueous ammonium formate (final concentration 10 mmol/L) and 0.1 vol% of formic acid as additives. Mobile phase B was a mixture of acetonitrile/isopropanol 5:2 (v/v) with the same additives. Gradient elution started at 50% mobile phase B, rising to 100% B over 40 minutes; 100% B were held for 10 minutes and the column was re‐equilibrated with 50% B for 8 minutes before the next injection. The flow rate was 150 μL/min, the samples were kept at 8°C and the injection volume was 2 μL. The mass spectrometer was operated in Data‐Dependent Acquisition mode using a HESI II ion source. Every sample was measured once in positive polarity and once in negative polarity. Full‐scan profile spectra were acquired in the Orbitrap mass analyzer at a resolution setting of 100 000 at *m*/*z* 400. For MS/MS experiments, the 10 most abundant ions of the full‐scan spectrum were sequentially fragmented. Data analysis was performed by Lipid Data Analyzer, a custom developed software tool described in.^36^, ^37^

### 2.8 Intestinal neutral lipid content

About 40‐50 mg of *terminal ileum (approximately 2 cm in front of the caecum)* of non‐fasted mice was homogenized in methanol/chloroform (2/1 v/v). Phase separation was achieved by addition of water. In brief, samples and lipid standards were added to a microtiter plate and incubated at 55°C for 20‐30 minutes. Isopropanol was added to the wells followed by a 30‐minute incubation step at 37°C. Fluorometric reagent was added and fluorescence was measured as Ex/Em = 490 nm/585 nm. Assay details: Fluorescent lipid assay kit for neutral lipids from Abcam (ab242307). Amount of neutral lipids was normalized to tissue weight.

### 2.9 Faecal non‐esterified fatty acid concentration

About 30‐40 mg faeces (of non‐fasted mice, collected at the end of the experiment from the individual mice) were homogenized in methanol/chloroform (2/1 v/v). Phase separation was achieved by addition of water. In brief, samples and fatty acid standards were added to a microtiter plate and incubated at 37°C for 30 minutes. Acyl‐CoA synthetase reagent was added to the wells followed by a 30‐minute incubation step at 37°C. Reaction reagent was added to the wells followed by a 30‐minute incubation step at 37°C (protected from light). Optical density was measured at 570 nm. Assay details: Fatty Acid Quantification Kit from abcam (ab65341). Non‐esterified fatty acid (NEFA) concentration was normalized to the amount of stool.

### 2.10 Bile acid profiling by ultra‐performance liquid chromatography‐tandem mass spectrometry

Profiles of murine primary and secondary unconjugated and conjugated C24‐BAs in liver and faeces were analysed as published previously^38^ on an Applied Biosystems AB SCIEX QTRAP 5500 platform. Unconjugated and taurine‐conjugated tetra‐ and pentahydroxylated BAs were identified from their molecular anions at *m/z* 463, 479, 530 and 546, respectively, and quantified in relation to D_4_‐CA and D_4_‐taurocholic acid (TCA).

### 2.11 Statistical analysis

Results were evaluated using SPSS V.23.0. Statistical analysis was performed using multifactorial ANOVA. Data are reported as means of seven to nine (WT Ctrl n = 9; BSEP KO Ctrl n = 7; WT MCD n = 9; BSEP KO MCD n = 8) animals per group ± SD. A *P* value ≤.05 was considered as statistically significant.

## 3 RESULTS

### 3.1 Alterations of BA metabolism and signalling in MCD‐fed BSEP KO mice

**Figure 1:**
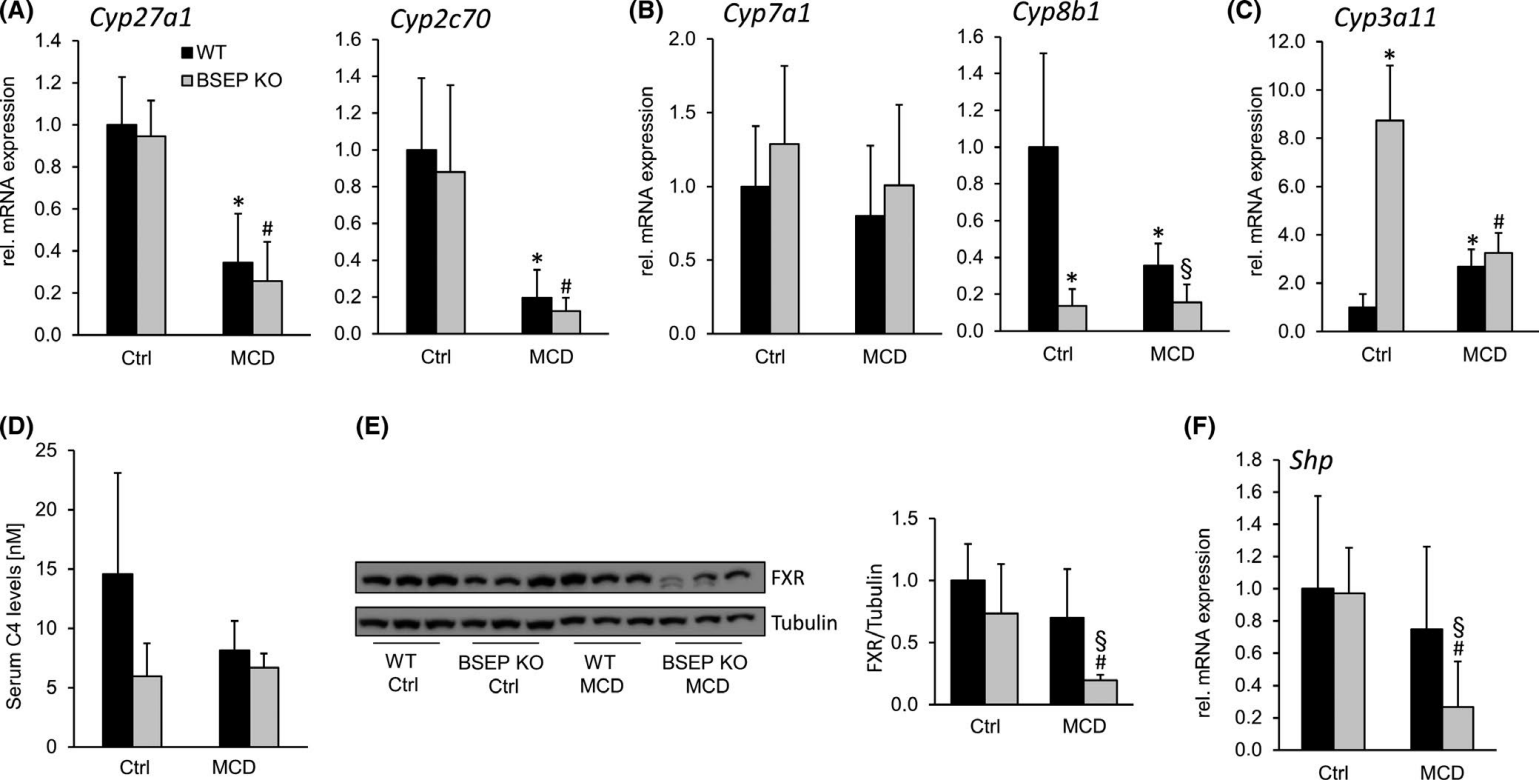
Bile acid metabolism is changed in BSEP KO mice challenged by MCD diet. (A) mRNA expression levels of *Cyp27a1*, *Cyp2c70* were reduced due to MCD feeding independent of the genotype. (B) While *Cyp7a1* levels remained unchanged at baseline as well as under MCD feeding, Cyp8b1 levels were reduced in BSEP KO mice at baseline and further decreased under MCD challenge. (C) *Cyp3a11* was increased in BSEP KO at baseline as was reduced MCD‐fed mice independent of the genotype. (D) Serum levels of 7‐hydroxy‐4‐cholesten‐3‐one (C4) did not differ significantly at baseline as well as under MCD challenge. (E) Western blot: Hepatic FXR protein levels were reduced in BSEP KO mice under MCD feeding (tubulin as loading control). (F) Hepatic mRNA expression of *Shp* was reduced in BSEP KO mice fed a MCD diet. * indicates a significant difference in untreated WT controls (Ctrl); § indicates a significant difference in MCD‐fed WT; # indicates a significant difference in BSEP KO Ctrl; *P* < .05

Since MCD feeding was shown to interfere with BA metabolism,^39^ recently found to be involved in progression of NASH,^20^ expression of key determinants of BA homeostasis, such as *Cyp27a1, Cyp2c70 *(alternative BA synthesis pathway) and *Cyp7a1, Cyp8b1* (classic BA synthesis pathway), serum levels of 7‐hydroxy‐4‐cholesten‐3‐one (C4) as marker of BA synthesis as well as expression of FXR (main BA sensor) and its downstream target *Shp* were investigated in our mouse model. Gene expression of *Cyp27a1* and *Cyp2c70* was reduced because of MCD feeding independent of the genotype (Figure 1A). While mRNA expression of *Cyp7a1* remained unchanged by MCD feeding (in line with unchanged C4 levels, Figure 1D), *Cyp8b1* levels were significantly reduced in BSEP KO mice at baseline as well as under MCD feeding (Figure 1B). In line, levels of TCA (endogenous FXR agonist) represented only 6%‐7% of total hepatic BA concentration in BSEP KO mice independent of MCD dietary feeding (Table 1), arguing for reduced FXR signalling in BSEP KO mice. Moreover, protein levels of FXR were reduced in MCD‐fed BSEP KO mice (Figure 1E). Accordingly, mRNA expression levels of *Shp* were reduced in BSEP KO MCD‐fed mice but remained unchanged in WT MCD‐fed mice (Figure 1F).

**Table 1:** Hepatic BA profile

pmol/mg	WT Ctrl	BSEP KO Ctrl	WT MCD	BSEP MCD
TαMCA	7.2 ± 6.8	34.9 ± 12.1^a^	17.2 ± 7.7	21.3 ± 3.7
TβMCA	71.0 ± 28.4	319.8 ± 91.8^a^	149.0 ± 54.8	531.9 ± 54.7^b^, ^c^
ToMCA	11.9 ± 6.5	56.6 ± 10.1^a^	8.5 ± 4.3	27.8 ± 2.5^b^, ^c^
TCA	50.4 ± 30.0	88.6 ± 37.6	223.8 ± 79.0^a^	74.1 ± 12.7^b^
TCDCA	1.0 ± 0.6	9.1 ± 0.1^a^	3.5 ± 1.2^a^	3.5 ± 0.5^c^
TDCA	1.4 ± 0.4	0.03 ± 0.02^a^	2.5 ± 0.7	0.1 ± 0.07^b^
TUDCA	2.0 ± 1.3	6.6 ± 3.4^a^	1.6 ± 0.6	2.4 ± 0.2
TLCA	0.1 ± 0.01	0.08 ± 0.01	0.04 ± 0.01^a^	0.03 ± 0.01^c^
αMCA	0.1 ± 0.01	1.0 ± 0.3^a^	0.05 ± 0.01^a^	0.1 ± 0.02^b^, ^c^
βMCA	0.5 ± 0.1	7.6 ± 0.8^a^	0.2 ± 0.1^a^	12.7 ± 2.4^b^, ^c^
oMCA	0.2 ± 0.1	0.4 ± 0.1	0.07 ± 0.02^a^	0.4 ± 0.1^b^
CA	0.2 ± 0 05	0.07 ± 0.03	0.2 ± 0.2	0.07 ± 0.04^b^
PHBA	2.0 ± 0.5	821.6 ± 270.0^a^	3.8 ± 2.5	361.3 ± 62.7^b^, ^c^
Total BAs	148.0 ± 73.5	1346.4 ± 363.8^a^	410.4 ± 149.6^a^	1035.7 ± 85.5^b^
TbMCA/Tot.BAs (%)	48.0 ± 19.2	23.7 ± 6.8^a^	36.2 ± 13.2	51.3 ± 5.3^b^, ^c^
TCA/Tot.BAs (%)	34.1 ± 20.3	6.6 ± 2.8^a^	54.4 ± 19.2	7.1 ± 1.2^b^
PHBA/Tot.BAs (%)	1.4 ± 0.4	61.0 ± 20.0^a^	0.9 ± 0.6	34.9 ± 6.0^b^, ^c^

Note

Data represent means ± SD.

^a^ Indicates a significant difference in untreated WT controls (Ctrl).

^b^ Indicates a significant difference in MCD‐fed WT.

^c^ Indicates a significant difference in BSEP KO Ctrl; *P* < .05.

Of note, despite reduced expression of *Cyp27a1 and Cyp2c70,* hepatic as well as faecal BA profiling revealed an increase in FXR antagonist TβMCA (Tables 1 and 2) in BSEP KO MCD‐fed mice. *Cyp3a11* (enzyme involved in BA detoxification), known to be increased in BSEP KO mice at baseline,^29^ was significantly repressed during MCD feeding (Figure 1C). Accordingly, hepatic levels of (poly)hydroxylated BAs dropped from 61% in BSEP KO Ctrl group to 35% in BSEP KO MCD‐fed mice (Table 1).

**Table 2:** Faecal BA profile

pmol/mg	WT Ctrl	BSEP KO Ctrl	WT MCD	BSEP MCD
TαMCA	2.8 ± 1.4	1.3 ± 0.9	2.1 ± 0.7	2.7 ± 1.9
TβMCA	38.4 ± 19.0	15.2 ± 12.4	32.9 ± 11.1	137.8 ± 54.3^b^, ^c^
ToMCA	6.7 ± 1.6	3.8 ± 2.2^a^	1.6 ± 0.5^a^	3.1 ± 1.9
TCA	18.9 ± 7.9	10.7 ± 8.2	37.9 ± 10.9	9.7 ± 4.3^b^
TCDCA	0.4 ± 0.1	1.2 ± 1.0	1.2 ± 0.7^a^	1.2 ± 0.6
TDCA	0.5 ± 0.1	n.d.	1.1 ± 0.6	n.d
TUDCA	0.8 ± 0.2	2.2 ± 1.6	0.7 ± 0.4	0.3 ± 0.2^c^
αMCA	33.8 ± 19.9	3.0 ± 1.5^a^	8.6 ± 5.4^a^	n.d
βMCA	341.6 ± 176.8	9.4 ± 5.8^a^	124.6 ± 69.1	12.9 ± 4.4^b^
oMCA	370.8 ± 79.0	21.9 ± 12.8^a^	22.7 ± 9.3^a^	3.2 ± 1.1^b^, ^c^
CA	105.3 ± 72.5	5.3 ± 2.4^a^	103.9 ± 90.4	3.4 ± 2.6^b^
CDCA	4.1 ± 4.0	2.0 ± 0.5	7.8 ± 4.0	6.0 ± 0.6^c^
DCA	242.1 ± 305.4	27.9 ± 19.7	91.7 ± 35.4	1.0 ± 0.02^b^, ^c^
UDCA	10.2 ± 7.5	2.9 ± 0.7	3.9 ± 2.1^a^	n.d.
LCA	4.7 ± 8.0	4.5 ± 2.7	2.6 ± 1.9^a^	n.d.
PHBA	22.8 ± 9.0	89.8 ± 13.2^a^	9.6 ± 2.5^a^	78.8 ± 28.2^b^
Total BAs	1337.0 ± 272.3	232.0 ± 72.0^a^	463.5 ± 223.5^a^	254.2 ± 77.8
TβMCA/Tot.BAs (%)	2.9 ± 1.2	6.5 ± 5.4	7.1 ± 2.4^a^	54.2 ± 21.3^b^, ^c^
TCA/Tot.BAs (%)	1.4 ± 1.3	4.6 ± 3.5	8.2 ± 2.3^a^	3.8 ± 1.7
PHBA/Tot.BAs (%)	1.7 ± 0.7	38.7 ± 5.7^a^	2.1 ± 0.5	31.0 ± 4.9^b^

Note

Data represent means ± SD.

^a^ Indicates a significant difference in untreated WT controls (Ctrl).

^b^ Indicates a significant difference in MCD‐fed WT.

^c^ Indicates a significant difference in BSEP KO Ctrl; *P* < .05.

### 3.2 Lipid metabolism is changed in MCD‐fed BSEP KO mice

**Figure 2:**
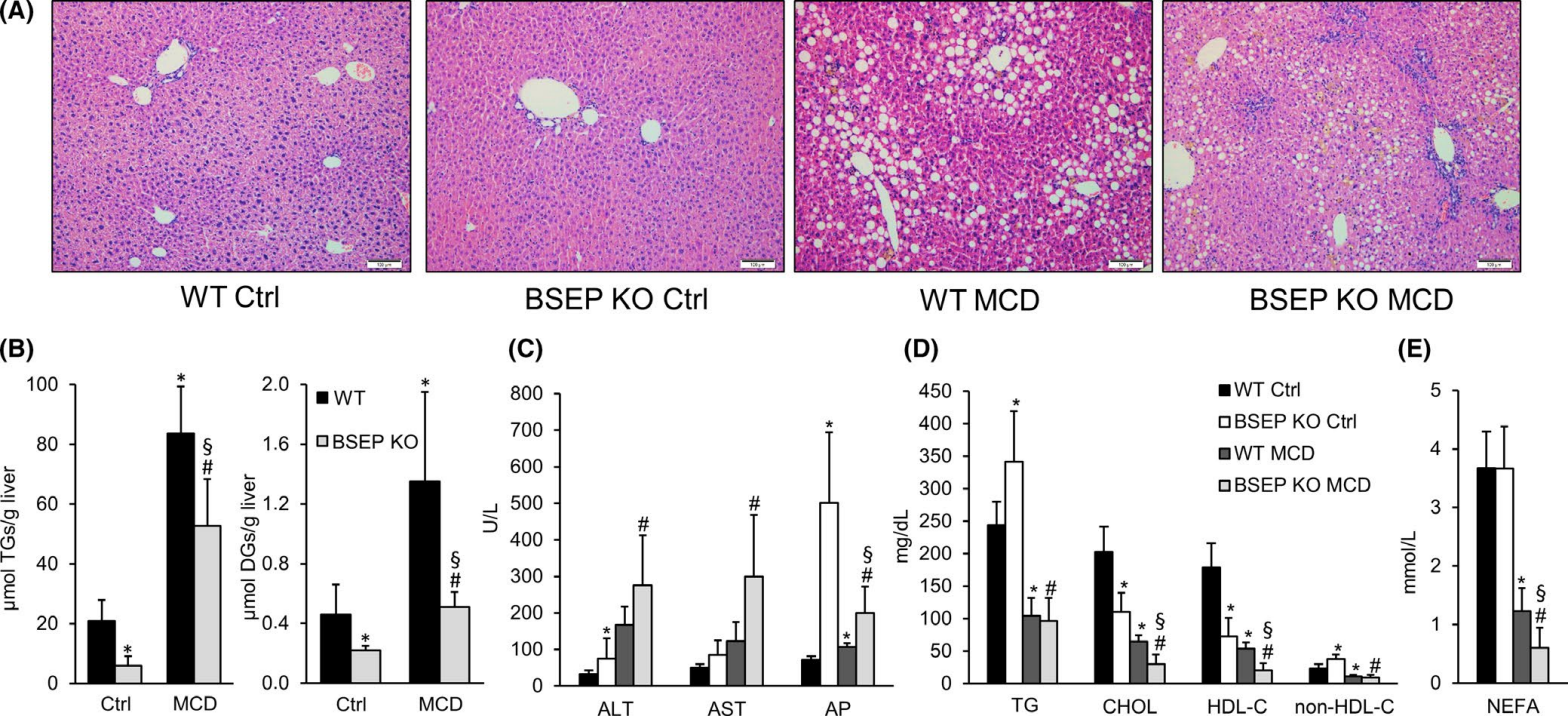
Methionine choline‐deficient (MCD) feeding‐induced liver injury in WT and BSEP KO mice. Wild‐type (WT) and BSEP knockout (KO) mice received methionine choline‐deficient (MCD) diet for 5 weeks. (A) H&E staining of liver sections of control and MCD‐fed WT and BSEP KO mice. Hepatic steatosis was evident in WT and less pronounced in BSEP KO mice challenged by MCD treatment (10× magnification, bar = 100 µm). (B) BSEP KO mice accumulated less hepatic diacylglycerols (DGs) and triglycerides (TGs) compared to WT mice under MCD feeding. (C) MCD diet‐induced liver injury as reflected by increased levels of liver enzymes (AST; ALT) in WT and BSEP KO mice. (D) Serum triglycerides (TG), cholesterol (CHOL), cholesterol fractions (HDL cholesterol and non‐HDL cholesterol) and (E) non‐esterified fatty acids (NEFAs) were significantly reduced after MCD feeding, with a more pronounced reduction in BSEP KO mice. Data represent means ± SD. *indicates a significant difference in untreated WT controls (Ctrl); § indicates a significant difference in MCD‐fed WT; # indicates a significant difference in BSEP KO Ctrl; *P* < .05

After 5 weeks of MCD feeding, liver histology as well as hepatic TG and diacylglycerol (DG) quantification revealed that BSEP KO mice, despite same food as well as caloric intake (Figure S1), accumulated less hepatic lipids than WT mice (Figure 2A,B). However, in MCD‐fed BSEP KO mice, serum levels of transaminases (ALT, AST) as well as AP were higher than in MCD‐fed WT mice (Figure 2C). Serum TGs were reduced by MCD feeding independent of the genotype (Figure 2D). Total and high‐density lipoprotein (HDL) cholesterol levels were reduced in MCD challenged WT and BSEP KO mice with a more pronounced reduction in BSEP KO mice (Figure 2D), while non‐HDL cholesterol fraction was reduced equally in WT and BSEP KO mice fed a MCD diet (Figure 2D). Non‐esterified fatty acids (Figure 2E) were also reduced in WT and BSEP KO mice fed with MCD diet, with a more evident difference in BSEP KO mice (Figure 2E).

### 3.3 Absence of BSEP results in impaired intestinal lipid absorption

**Figure 3:**
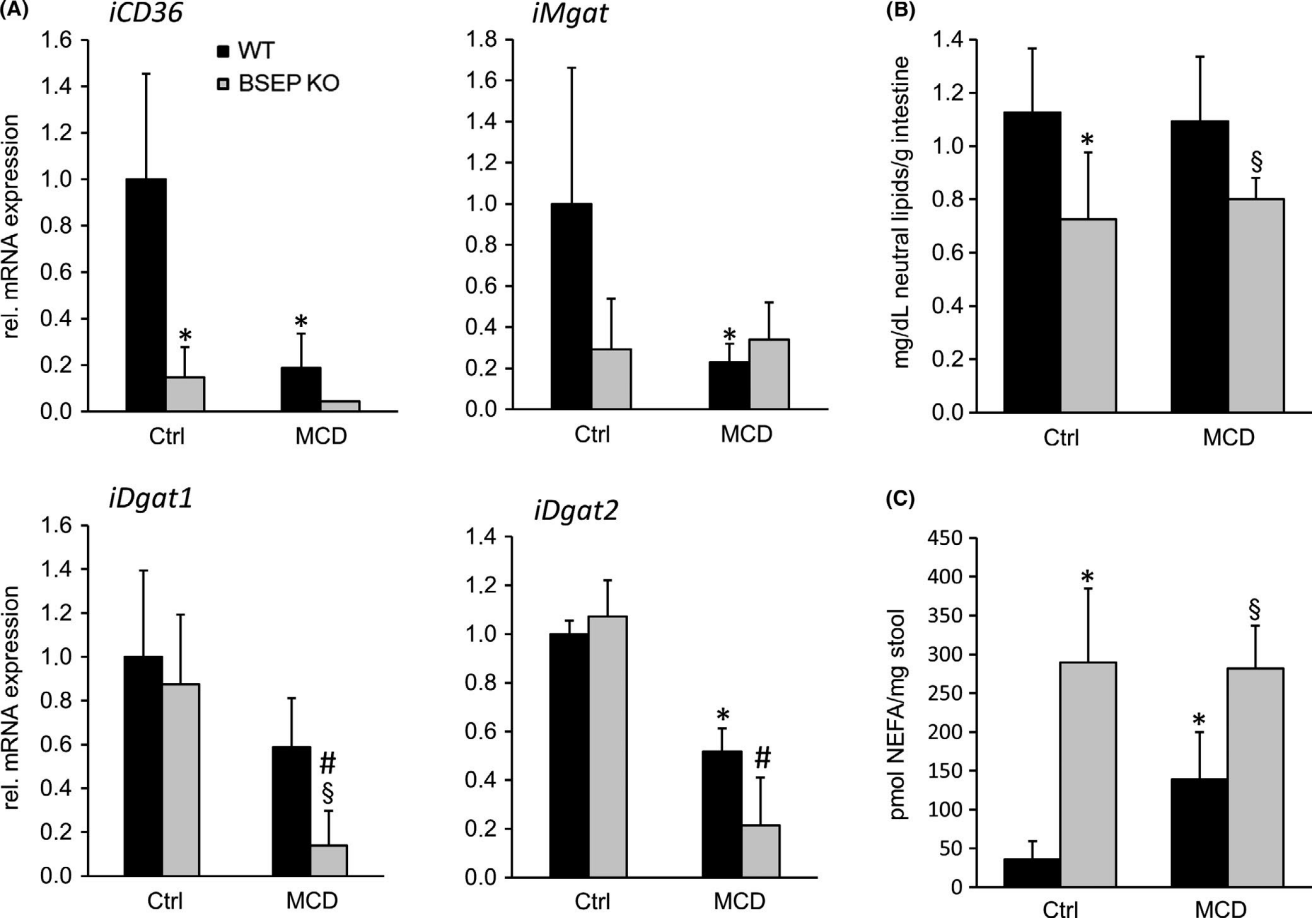
Absence of bile salt export pump (BSEP) results in changes in hepatic and faecal lipid composition in MCD‐fed mice. (A) mRNA expression of genes/enzymes involved in intestinal lipid metabolism was investigated. *CD36* (fatty acid uptake) mRNA levels were reduced in BSEP KO mice at baseline and dropped further under MCD feeding. While expression levels of *Mgat* were reduced due to MCD feeding to the same extent in WT and BSEP KO mice, mRNA levels of *Dgat1* and *Dgat2* were significantly lower in BSEP KO MCD mice compared to challenged WT animals. (B) Neutral lipid storage assay revealed reduced lipid content in intestine of BSEP KO mice at baseline as well as under MCD feeding. (C) Faecal non‐esterified fatty acid (NEFA) concentrations were increased in BSEP KO mice at baseline and after MCD feeding compared to WT mice. Data represent means ± SD. *Indicates a significant difference in untreated WT controls (Ctrl); § indicates a significant difference in MCD‐fed WT; # indicates a significant difference in BSEP KO Ctrl; *P* < .05

To investigate whether differences in hepatic lipid metabolism in MCD‐fed mice may result from impaired intestinal lipid absorption in BSEP KO mice, intestinal lipid metabolism was investigated. Gene expression profiling revealed reduced expression of intestinal *CD36* (FA uptake) in BSEP KO mice already at baseline. MCD feeding reduces the expression levels even more. Accordingly, expression of *Mgat* and *Dgat1* and *2* (enzymes involved in TG formation) was reduced due to MCD feeding. In case of *Dgat1* and *Dgat2*, reduction was even more pronounced in BSEP KO MCD‐fed mice (Figure 3A). In line, intestinal neutral lipid content was reduced in BSEP KO mice at baseline as well as under MCD challenge (Figure 3B). Subsequently, faecal NEFA concentrations were measured. BSEP KO mice at baseline as well as under MCD conditions displayed markedly elevated amounts of NEFA in stool (Figure 3C). Together these findings, indicate that BSEP KO mice may have reduced lipid absorption, resulting in less lipid accumulation in the liver.

### 3.4 Hepatic FA metabolism is altered in MCD‐fed BSEP KO mice

**Figure 4:**
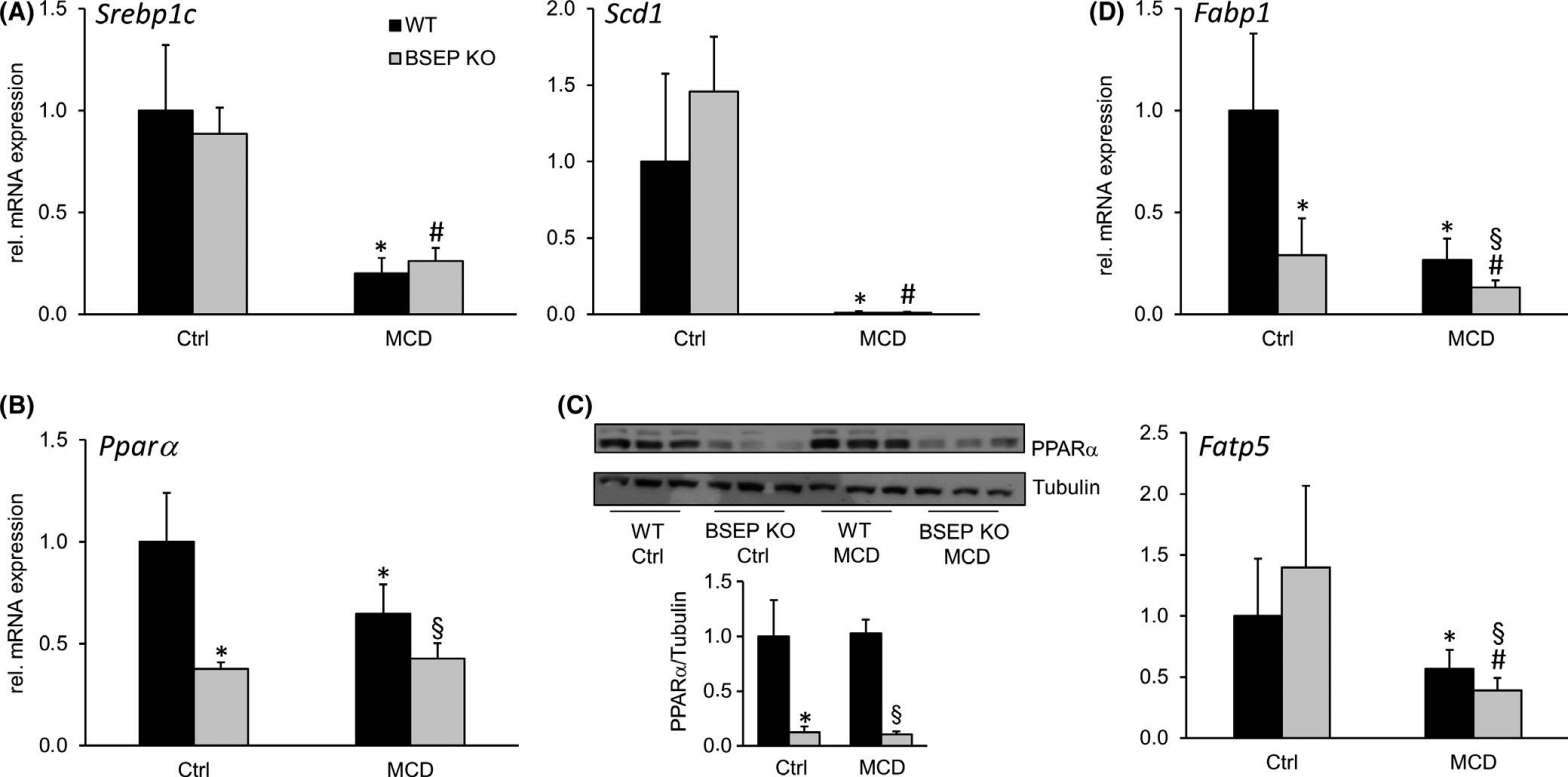
Loss of BSEP leads to changes of key regulators of hepatic lipid metabolism. Markers for (A) de novo lipogenesis: *Srebp1c* and *Scd1*, (B and C) β‐oxidation PPARα and (D) fatty acid transporters *Fatp5* and *Fabp1* were determined by quantitative polymerase chain reaction (qPCR) and western blotting. (A) de novo lipogenesis markers, *Srebp1c* and *Scd1*, were reduced by MCD feeding independent of the genotype. (B) mRNA and (C) protein expression of PPARα were reduced in BSEP KO mice at baseline as well as under MCD feeding when compared to WT mice. (D) mRNA levels of *Fabp1* and *Fatp5* were reduced by MCD feeding to a higher extent in BSEP KO than WT mice. *Indicates a significant difference in untreated WT controls (Ctrl); § indicates a significant difference in MCD‐fed WT; # indicates a significant difference in BSEP KO Ctrl; *P* < .05

To further explore whether differences in lipid accumulation after MCD feeding might be also influenced by differences in de novo lipogenesis, FA β‐oxidation and/or FA uptake and transport, we measured the mRNA expression of hepatic de novo lipogenesis markers *Srebp1c*, *Scd1* (Figure 4A), β‐oxidation master regulator *PPARα*, (Figure 4B,C) as well as *Fatp5* (FA uptake*)* and *Fabp1* (intracellular FA transport) (Figure 4D). Genes involved in de novo lipogenesis were repressed by MCD feeding independent of the BSEP genotype (Figure 4A). Notably, *PPARα* expression was already significantly reduced in BSEP KO mice at baseline at both mRNA and protein levels. This reduction compared to WT mice was maintained under MCD challenge (Figure 4B,C). PPARα downstream targets, *Fabp1* and *Fatp5*, were significantly lower in MCD‐fed BSEP KO mice compared to their control group and MCD‐fed WT mice. Of note, *Fabp1* expression was also significantly reduced in BSEP KO mice at baseline (following the PPARα expression levels). These findings suggest that in addition to impaired lipid absorption also reduced FA transport and uptake could account for differences in hepatic lipid content.

### 3.5 Hepatic inflammation is aggravated in MCD‐fed BSEP KO mice

**Figure 5:**
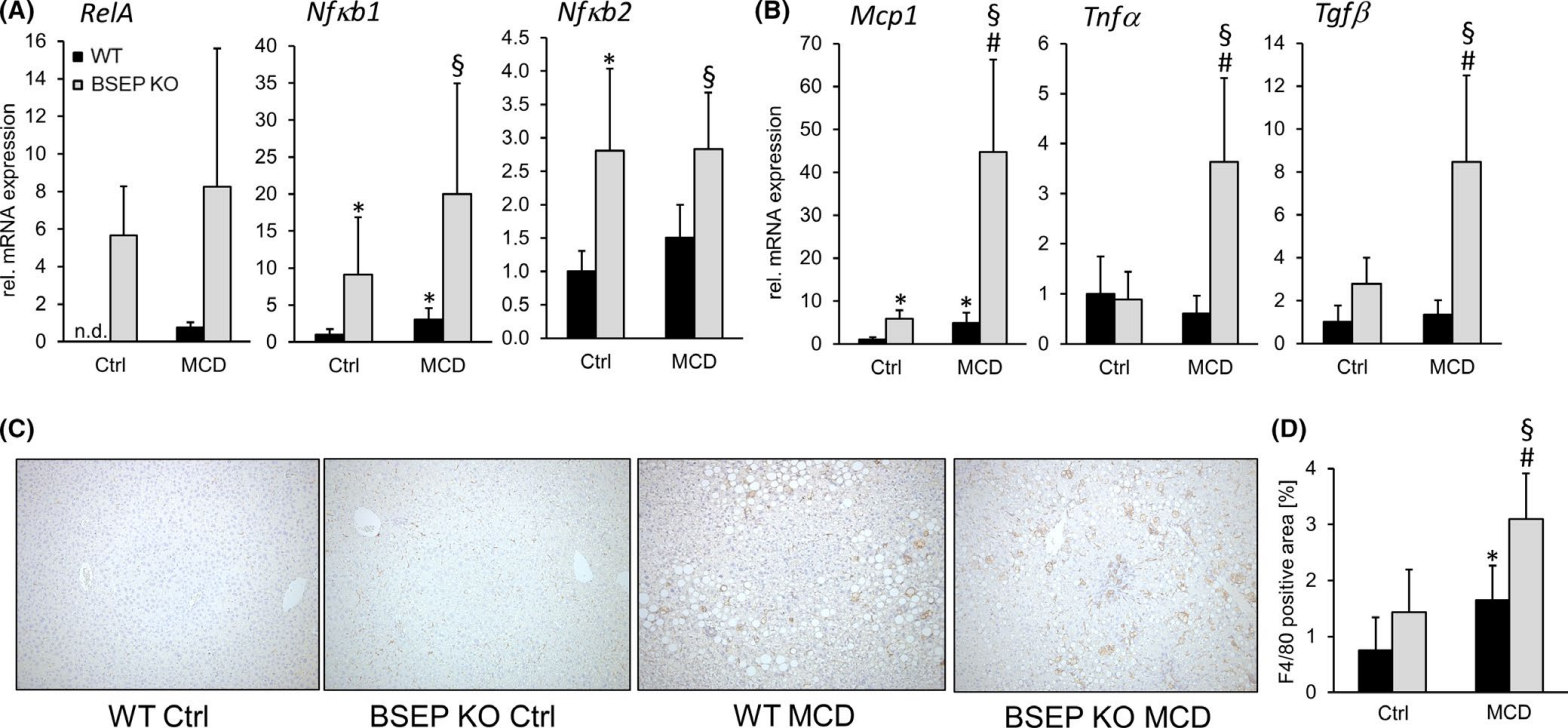
Loss of BSEP aggravates hepatic inflammation under MCD feeding. (A) Expression of NFκB subunits, *RelA, Nfκb1* and *Nfκb2*, was assessed by qPCR. All genes were increased in BSEP KO mice at baseline and tended to be further enhanced by MCD feeding. (B) Inflammatory markers, *Mcp1, Tnfα* and *Tgfβ*, were assessed by qPCR. Expression of all genes was markedly increased in MCD‐fed BSEP KO mice. (C) Representative immunohistochemistry for F4/80^+^ cells of liver specimens of control and MCD‐fed WT and BSEP KO mice (10× magnification) shows increased inflammation in MCD‐fed BSEP KO mice. This observation was confirmed by computational quantification of the sections (D) BSEP KO MCD‐fed mice show the highest levels of F/80 positive area. *Indicates a significant difference in untreated WT controls (Ctrl); § indicates a significant difference in MCD‐fed WT; # indicates a significant difference in BSEP KO Ctrl; *P* < .05

Improved steatosis in the presence of elevated liver enzymes points towards a possible dissociation between attenuated steatosis and aggravation of hepatocellular injury. Also, reduced PPARα protein expression already at baseline in BSEP KO mice (Figure 4C) could be interpreted as a hint towards increased susceptibility to inflammation. Since the inflammatory mediator NFκB is known to be regulated by PPARα,^40^ expression of NFκB subunits *RelA, Nfκb1* and *Nf*k*b2* was investigated (Figure 5A). Expression levels of all genes were increased in BSEP KO mice already at baseline and tended to be further increased under MCD conditions. Additionally, mRNA expression of *Mcp1*, *Tnfα* and *Tgfβ* was markedly increased in BSEP KO mice fed a MCD diet compared to WT mice (Figure 5B). In line, F4/80 IHC revealed an increase of F4/80 positive cells in MCD‐fed BSEP KO mice compared to challenged WT mice (Figure 5C,D). To exclude the possibility that inflammation might result from modifications in endoplasmatic reticulum (ER) stress, we also measured mRNA expression of the ER stress markers *ErDj4* and *Grp78* (Figure S2). Expression levels of these genes did not differ between WT and BSEP KO MCD‐fed mice. Since progression of NAFLD may also comprise development of fibrosis, Sirius Red staining was performed and mRNA and protein expression of several markers, such as *Col1a1*, *Col1a2*, αSMA and hydroxyproline, were investigated (Figure S3). However, development of fibrosis under MCD conditions was only mild and did not depend on the presence or absence of BSEP and subsequent changes in BA signalling.

## 4 DISCUSSION

**Figure 6:**
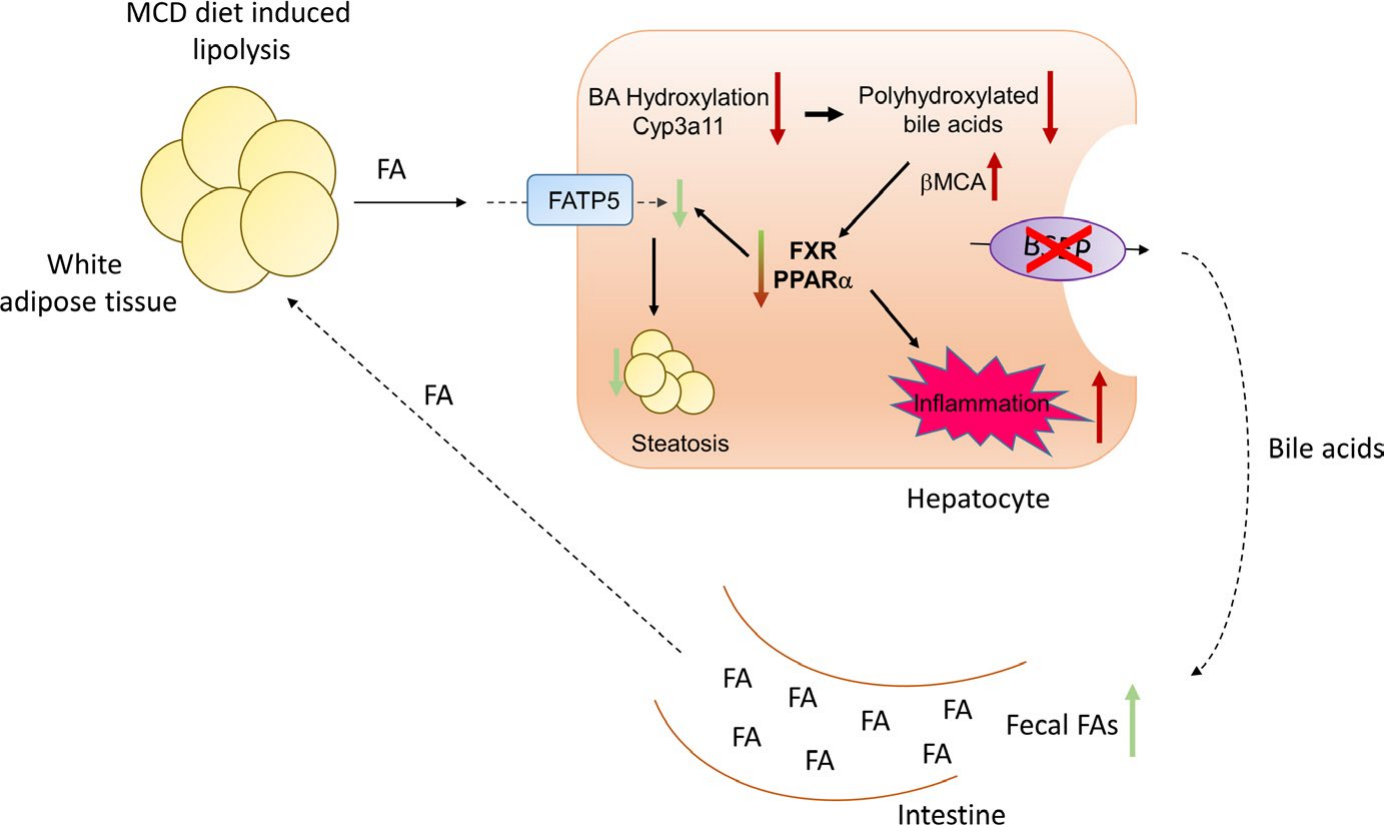
Proposed mechanism how absence of BSEP impacts on hepatic lipid metabolism and inflammation. Impaired enterohepatic circulation of bile acids (BAs) in BSEP KO mice increases faecal FA excretion at baseline as well as under MCD conditions. Additionally, BSEP KO mice have a more hydrophilic BA pool mainly consisting of PHBA, which is a disadvantage in term for micelle formation. Moreover, low expression levels of PPARα in BSEP KO mice (at baseline and under MCD feeding) result in reduced hepatic FA uptake further contributing to improved steatosis. MCD feeding reduces hydroxylation/detoxification of BAs and favours formation of βMCA, a BA exerting FXR antagonistic function. Reduced PPARα together with reduced FXR signalling may account for the increased inflammation seen in BSEP KO mice. Red arrows indicate a disease worsening effect; green arrows indicate a disease improving effect

In this study, we examined the impact of increased levels of hydrophilic BAs in the liver on the development of fatty liver and its progression to NASH (Figure 6). To this purpose, BSEP KO mice were challenged with MCD diet to induce steatohepatitis, a model which has been used previously by others to induce profound steatosis and inflammation and some degree of fibrosis in rodents,^41^, ^42^, ^43^, ^44^, ^45^ although several short comings such as weight loss, lack of insulin resistance and obesity in this model need to be acknowledged.^46^, ^47^ However, the lack of methionine and choline makes patients more susceptible to develop fatty liver.^48^, ^49^ Moreover, MCD diet increases lipolysis in adipose tissue and subsequently the shift of FAs from adipose tissue to the liver.^46^ Therefore, MCD diet is a useful model to investigate how the liver can cope with FA overflow. More specifically in this project, we investigated how changes in hepatic BA pool (as it is seen in BSEP KO mice) interfere with FA spillover from the periphery. To this purpose, BSEP KO mice were challenged with MCD diet for 5 weeks. BSEP KO mice subjected to MCD diet developed severe inflammation despite milder hepatic steatosis.

Notably, a dissociation between hepatic steatosis and inflammation was also seen in MCD‐fed WT mice where hepatic TG synthesis was inhibited via DGAT2 antisense oligonucleotide.^50^ As a result of elevated hepatic free FA content with subsequent lipotoxicity, oxidative damage, liver inflammation and fibrosis were aggravated while hepatic steatosis was reduced.^50^ Low mRNA expression of *Fatp5* and *Fabp1* in MCD‐fed BSEP KO mice revealing reduced FA uptake and intracellular FA transport may also explain, at least in part, the decreased hepatic lipid accumulation in MCD‐fed BSEP KO mice. An intact enterohepatic circulation of BAs is key for physiologic intestinal lipid absorption, and interruption of BA circulation between liver and intestine results in steatorrhea.^51^ BSEP KO mice have a severely diminished enterohepatic circulation and it was impossible to measure bile flow. This finding was in line with low biliary pressure found in BSEP KO mice after 7 days of bile duct ligation.^29^ Moreover, BSEP KO mice have a more hydrophilic (poorer micelle forming) BA composition with polyhydroxylated bile acids (PHBA), which were, however, reduced by MCD feeding. In line, BSEP KO mice showed reduced intestinal lipid content and high faecal NEFA levels already at baseline arguing for potentially impaired lipid absorption in these mice. Reduced lipid absorption leading to lack of PPARα ligands (long‐chain FAs, such as arachidonic acid, have been shown to serve as endogenous PPARα ligands^52^) together with reduced expression of this key regulator of FA oxidation and inflammation may (at least in part) contribute to increased inflammation seen in BSEP KO mice under MCD feeding. Moreover, PPARα is also known to function as suppressor of the inflammatory mediator NFκB;^40^ in line with reduced PPARα expression in BSEP KO mice at baseline, expression levels of NFκB subunits *RelA, Nfκb1* and *Nfκb2* are markedly increased in these animals, further arguing that BSEP KO mice are more susceptible to the development of hepatic inflammation. MCD feeding reduces hydroxylation/detoxification of BAs and favours formation of βMCA, a BA exerting FXR antagonistic function. Thus, reduced PPARα together with reduced FXR signalling may account for the increased inflammation seen in BSEP KO mice.

Under MCD conditions, lack of BSEP leads to changes in hepatic BA composition favouring TβMCA (51% in BSEP MCD compared to 23% in BSEP KO control animals), known to have FXR antagonistic properties.^53^ Furthermore, the endogenous FXR agonist TCA represents only 6%‐7% of the hepatic BA pool in BSEP KO mice at baseline as well as under MCD feeding. Changes in BA composition possibly leading to reduced FXR function in BSEP MCD‐fed mice may also contribute to increased hepatic inflammation in these animals. Since besides its regulatory function in lipid and BA metabolism, FXR has anti‐inflammatory actions via inhibition of the nuclear factor kappa‐light‐chain‐enhancer of activated B cells (NFκB) signalling pathway^54^, ^55^ and activation of suppressor of cytokine signalling (Socs) 3.^56^

Based on the tremendous increase of tauroβmuricholicacid (TbMCA) concentration in liver and stool of MCD‐fed BSEP KO mice, expression levels of enzymes involved in the synthesis pathway should be elevated. However, mRNA expression of Cyp27a1 and Cyp2c70 was significantly decreased for both genes under MCD condition independent of the genotype. This finding is in line with previous observations.^39^, ^57^, ^58^ It has been shown that despite elevated bMCA concentration in serum, enzymes involved in their synthesis are downregulated under MCD conditions. Absence of choline and subsequent increase of proinflammatory cytokine expression/secretion from hepatocytes are responsible for reduced expression of BA synthesis genes while at the same time these cytokines interfere with BA transporter expression resulting in retention of BAs, thereby explaining increased levels of MCA despite reduced expression levels of enzymes involved in their synthesis. Among several Cyps involved in BA synthesis, Cyp7a1 was one of the few not being reduced due to MCD feeding.^39^, ^57^, ^58^ Furthermore, the fact that Cyp7a1 expression does not show the expected increase might result from changes in intestinal FXR‐FGF15 signalling. Since faecal BA profiling penalizes FXR signalling, we are tempted to speculate that subsequent FGF15 signalling from intestine to the liver is also reduced. SHP mRNA expression could be reduced due to MCD feeding even in the absence of reduced intrahepatic BA content. This might be due to a disruption of BA homeostasis during the course of NASH where proinflammatory cytokines and cellular stress may disrupt BA homeostasis, causing a vicious cycle.^39^, ^57^, ^58^

About 50%‐60% of hepatic BAs in BSEP KO mice at baseline are PHBA and,^29^ which is in line with increased expression levels of enzymes involved in BA detoxification and.^29^ Metabolic preconditioning with a hydrophilic BA pool is supposed to protect BSEP KO mice from cholestasis‐induced injury, which is reflected by the absence of hepatic inflammation and fibrosis after bile duct ligation and 3,5‐diethoxycarboncyl‐1,4‐dihydrocollidine (DDC) feeding.^29^ However, MCD feeding reduced the expression of Cyp3a11 (PXR target gene) and hepatic levels of PHBA by about 30%, suggesting that MCD feeding impaired BA hydroxylation/detoxification in BSEP KO mice. Thereby, the effects of metabolic preconditioning in BSEP KO mice were blunted. The importance of a hydrophilic BA pool in defence against the development of steatohepatitis is underlined by the very hydrophobic BA pool found in mice with BSEP overexpressing that under lithogenic diet conditions developed only mild steatosis but severe hepatitis.^27^

In summary, our data show that altered BA composition and signalling can result in dissection of hepatic lipid accumulation and inflammation, potentially by changing lipid absorption as well as FXR and PPARα signalling as key regulators of hepatic metabolism and inflammation. Further studies are required to fully understand the role of BA metabolism and signalling as potential pathogenetic factors in the multihit pathogenesis and progression of NASH, as well as the role as biomarkers and therapeutic targets.

## CONFLICT OF INTEREST

Michael Trauner served as a consultant for Albireo, BiomX, Boehringer Ingelheim, Falk, Genfit, Gilead, Intercept, MSD, Novartis, Phenex and Regulus, and is a member of the speakers’ bureau of Falk, Intercept, Gilead, MSD and Roche. He further received travel grants from Abbvie, Falk, Gilead, Intercept and Roche and unrestricted research grants from Albireo, Cymabay, Falk, Gilead, Intercept MSD and Takeda. He is also coinventor of a patent on the medical use of nor‐UDCA filed by the Medical University of Graz. All other authors have no financial disclosures concerning this study to report. The other authors have no conflict of interest.

## AUTHOR CONTRIBUTIONS

CDF: writing of the manuscript, data collection, statistical analysis and interpretation of data; SK, DS, VM, AW, MS, TC, HS: data collection, critical revision of the manuscript for important intellectual content; TS, HUM: critical revision of the manuscript for important intellectual content; MT: study concept and design, interpretation of data, outlining and revising the manuscript.

## Supporting information

Figure S1

Figure S2

Figure S3

Table S1
