# Supplementary figures and images for: Absence of Bsep/Abcb11 attenuates MCD diet‐induced hepatic steatosis but aggravates inflammation in mice
Source: Liver Int. 2020 Mar 18;40(6):1366–77. doi: 10.1111/liv.14423 (PMC7317533; doi:10.1111/liv.14423)

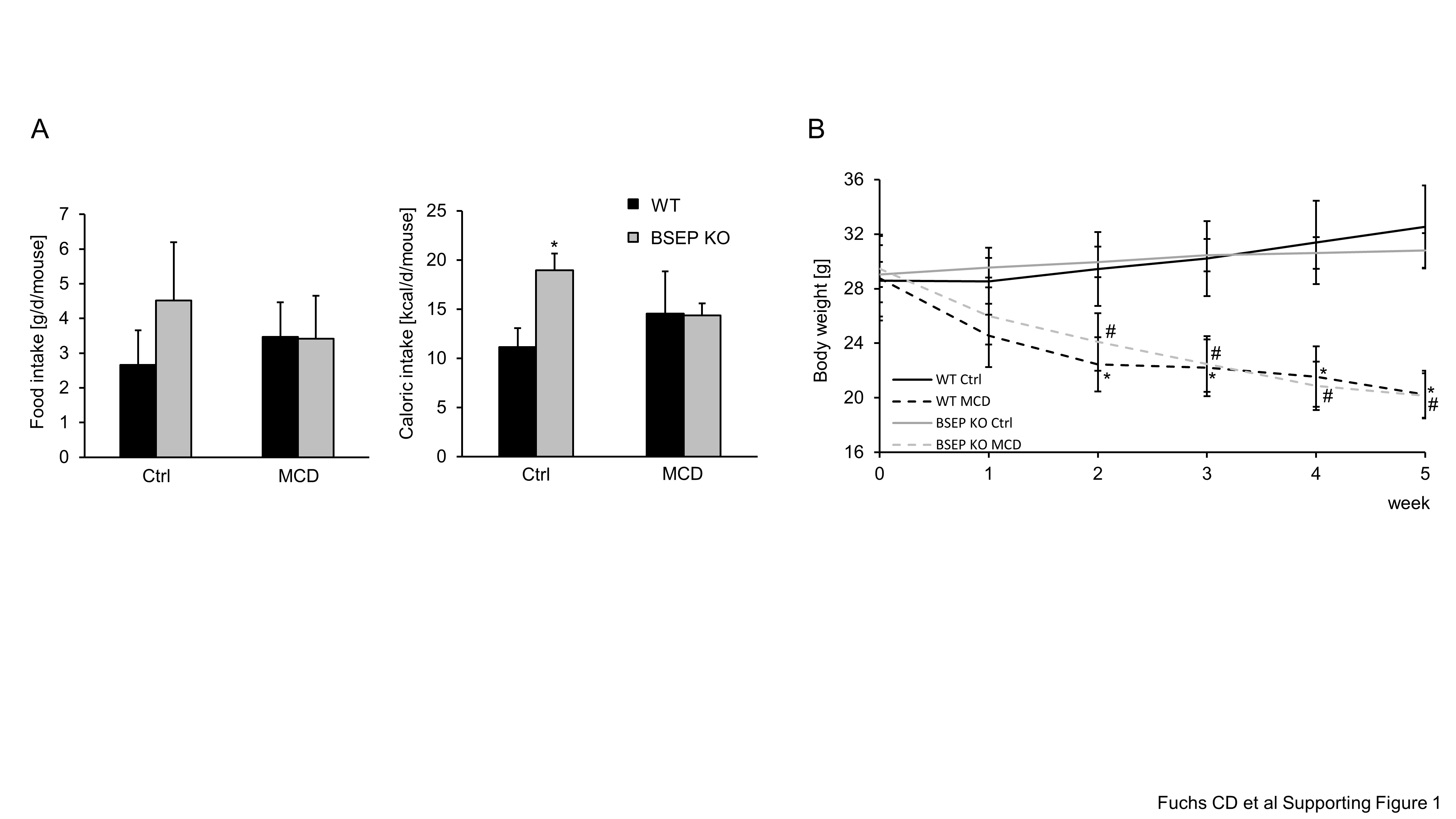

Supplement: Supplementary file 1 — Figure S1 [file LIV-40-1366-s001.TIF]

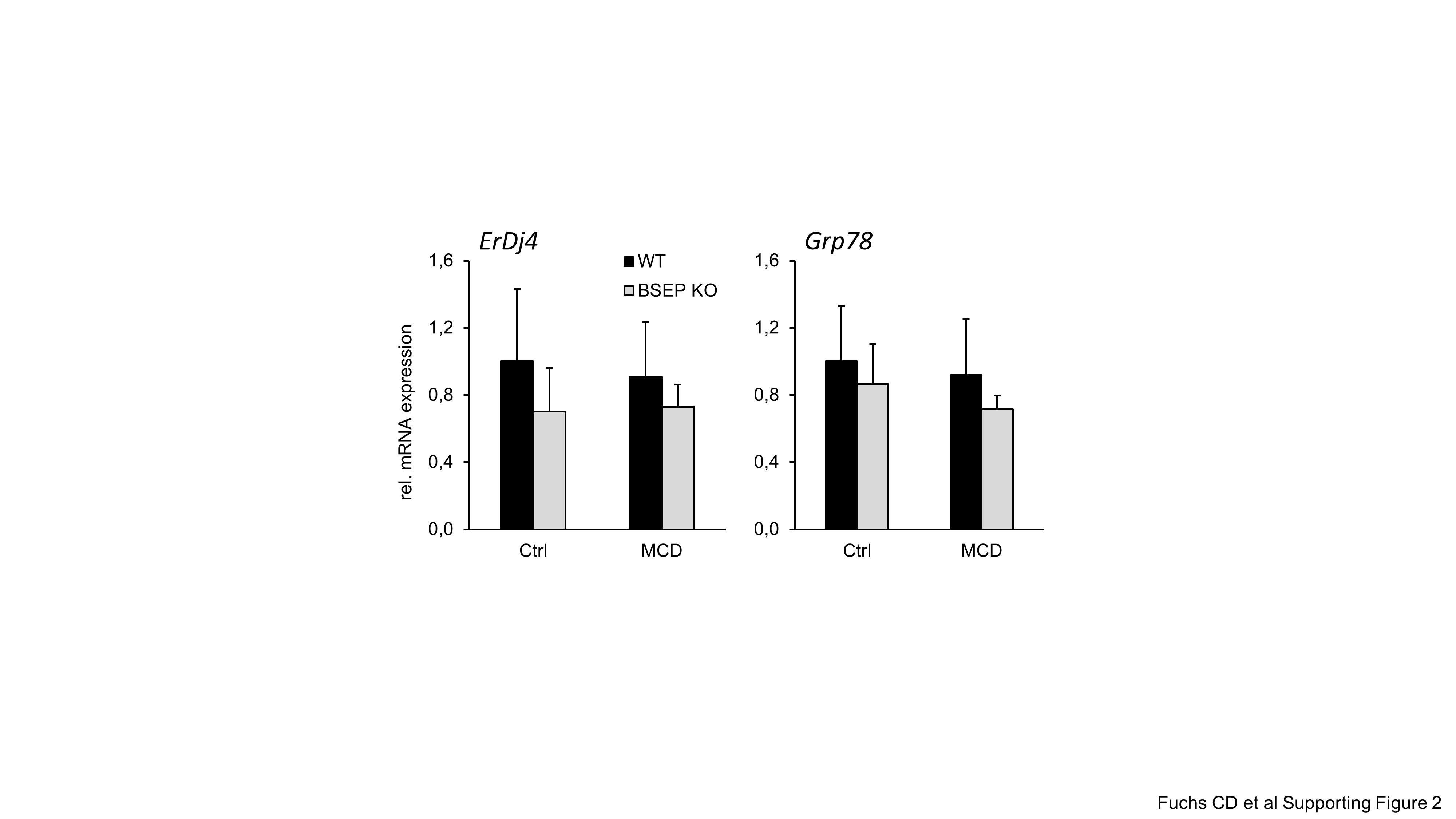

Supplement: Supplementary file 2 — Figure S2 [file LIV-40-1366-s002.TIF]

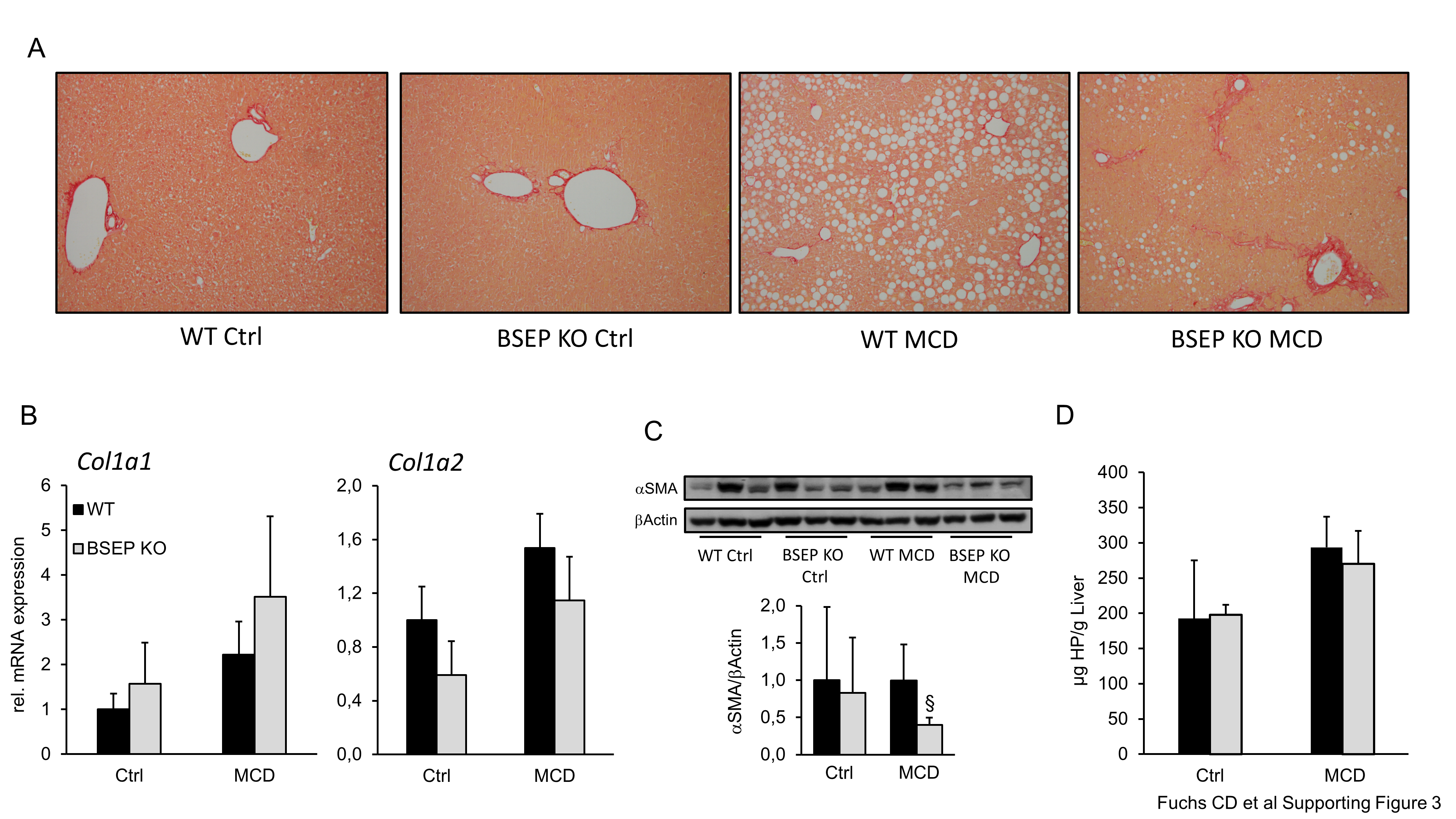

Supplement: Supplementary file 3 — Figure S3 [file LIV-40-1366-s003.TIF]
