# Supplementary material for: Absence of Bsep/Abcb11 attenuates MCD diet‐induced hepatic steatosis but aggravates inflammation in mice
Source: Liver Int. 2020 Mar 18;40(6):1366–77. doi: 10.1111/liv.14423 (PMC7317533; doi:10.1111/liv.14423)
Supplement: Supplementary file 4 — Table S1 [file LIV-40-1366-s004.docx]

**Supporting material**

**Absence of Bsep/Abcb11 attenuates MCD-diet induced hepatic steatosis but aggravates inflammation in mice**

Claudia Daniela Fuchs^1^, Sebastian Krivanec^1^, Daniel Steinacher^1^, Veronika Mlitz^1^, Annika Wahlström^2^, Marcus Stahlman^2^, Thierry Claudel^1^, Hubert Scharnagl^3^, Tatjana Stojakovic^4^, Hanns-Ulrich Marschall^2^, and Michael Trauner^1^

^1^Hans Popper Laboratory of Molecular Hepatology, Division of Gastroenterology and Hepatology, Department of Internal Medicine III, Medical University of Vienna, Austria

^2^Sahlgrenska Academy, Institute of Medicine, Department of Molecular and Clinical Medicine/Wallenberg Laboratory, University of Gothenburg. Gothenburg, Sweden

^3^Clinical Institute of Medical and Chemical Laboratory Diagnostics, Medical University of Graz, Austria

^4^Clinical Institute of Medical and Chemical Laboratory Diagnostics, University Hospital Graz, Austria

### Corresponding author:

Michael Trauner, MD

Professor and Chair of Gastroenterology and Hepatology

Division of Gastroenterology and Hepatology, Department of Internal Medicine III, Medical University of Vienna, Waehringer Guertel 18-20, A-1090 Vienna, Austria.

Tel: +43 1 40 40047410

Email: michael.trauner@meduniwien.ac.at

This work was supported by the grants F3517-B20, F3008-B05 and F7310-B21 from the Austrian Science Foundation (to MT).

**Supporting Figure Legends**

**Supporting Figure 1: MCD feeding does not interfere with food intake but reductesbody weight.** (A) While food intake as well as caloric intake tended to be increased in BSEP KO mice fed a MCD diet, no differences were seen in groups treated with MCD diet. (B) MCD feeding lowered body weight independent of the genotype.

**Supporting Figure 2: MCD feeding has no impact on development of ER stress and is independent from presence of BSEP.** qPCR was used to determine mRNA expression levels of ER stress markers *ErDj4* and *Grp78*. Both the genes remained unaffected by MCD feeding.

**Supporting Figure 3: WT and BSEP KO mice subjected to MCD diet do not develop significant hepatic fibrosis.** (A) Sirius red staining of liver sections of control and MCD-fed WT and BSEP KO mice. Induction of fibrosis by MCD feeding is only marginal. (B) Gene expression profile of fibrotic markers Col1a1 and Col1a2 showed only a mild trend for an increase by MCD feeding. (C) αSMA protein expression remained unchanged between control and MCD-fed animals. (D) Hydroxyproline (HP) levels remained also unaffected by MCD feeding.

**Supporting Table 1: Primer Sequences**

| **Gene** | **Forward primer** | **Reverse primer** |
| --- | --- | --- |
| **TNFα** | cat ctt ctc aaa att cga gtg aca a | tgg gag tag aca agg tac aac cc |
| **MCP-1** | ggc tgg aga gct aca aga gg | atg tct gga ccc att cct tc |
| **TGF-β** | tga cgt cac tgg agt tgt acg g | ggt tca tgt cat gga tgg tgc |
| **PPARα** | gtg gct gct ata att tgc tgt g | gaa ggt gtc atc tgg atg g |
| **FATP5** | gca tgg cgt aac agt gat ct | ttg tct tct ggt tgc tca gg |
| **FABP1** | ggg aag aaa atc aaa ctc acc atc | agt tgt cac cat ttt att gtc acc |
| **Cyp27a1** | gca caa gga agt gac tgg tg | ctg tga tga tcc ggg agt ttg t |
| **Cyp2c70** | tgg gct ttt gct cct gct gaa g | tca gtg tac ggc atg tgg ttc c |
| **Cyp7a1** | cag gga gat gct ctg tgt tca | agg cat aca tcc ctt ccg tga |
| **Cyp8b1** | tta agg ctg gct tcc tga gc | tcg acg gaa ctt cct gaa cag |
| **Cyp3a11** | cca cca gta gca cac ttt cc | ttc cat ctc cat cac agt atc a |
| **Shp** | aag ggc acg atc ctc ttc aa | gta cca ggg ctc caa gac t |
| **CD36** | gat cgg aac tgt ggg ctc at | ggt tcc ttc ttc aag gac aac ttc |
| **Mogat** | acc aca aat cct gcg aaa gg | cca tca tgg ttc tac ccg ga |
| **Dgat1** | ggt gcc ctg aca gag cag at | cag taa ggc caca agc tgc tg |
| **Dgat2** | ggg tcc aga aga agt tcc aga ag | ccc agg tgt cag agg aga aga g |
| **Srebp1c** | gga gat gct atc tcc atg gca | gaa acg tgt caa gaa gtg cag g |
| **Scd1** | atg ctc caa gag atc tcc agt tct | ctt cac ctt ctc tcg ttc att tcc |
| **RelA** | gca gct acg gcg gcc ttc tg | ggt ggc gat cat ctg tgt ctg gc |
| **NfkB1** | gga gac cgg caa ctc aca gac ag | aca tga ggc gca cca cgc tc |
| **NfkB2** | cag acg cgc gtg ctc tgc ta | gtg cag tgg cgt gtc tcc gt |
| **36b4** | gct tca ttg tgg gag cag aca | cat ggt gtt ctt gcc cat cag |
